# A New Experimental Model for Assessing Drug Efficacy against *Trypanosoma cruzi* Infection Based on Highly Sensitive In Vivo Imaging

**DOI:** 10.1177/1087057114552623

**Published:** 2015-01

**Authors:** Michael D. Lewis, Amanda Fortes Francisco, Martin C. Taylor, John M. Kelly

**Affiliations:** 1 Department of Pathogen Molecular Biology, London School of Hygiene and Tropical Medicine, London WC1E 7HT, UK

**Keywords:** Chagas disease, trypanosomes, drugs, imaging

## Abstract

The protozoan *Trypanosoma cruzi* is the causative agent of Chagas disease, one of the world’s major neglected infections. Although development of improved antiparasitic drugs is considered a priority, there have been no significant treatment advances in the past 40 years. Factors that have limited progress include an incomplete understanding of pathogenesis, tissue tropism, and disease progression. In addition, in vivo models, which allow parasite burdens to be tracked throughout the chronic stage of infection, have been lacking. To address these issues, we have developed a highly sensitive in vivo imaging system based on bioluminescent *T. cruzi*, which express a red-shifted luciferase that emits light in the tissue-penetrating orange-red region of the spectrum. The exquisite sensitivity of this noninvasive murine model has been exploited to monitor parasite burden in real time throughout the chronic stage, has allowed the identification of the gastrointestinal tract as the major niche of long-term infection, and has demonstrated that chagasic heart disease can develop in the absence of locally persistent parasites. Here, we review the parameters of the imaging system and describe how this experimental model can be incorporated into drug development programs as a valuable tool for assessing efficacy against both acute and chronic *T. cruzi* infections.

## Background

The protozoan *Trypanosoma cruzi* is the causative agent of Chagas disease and is the most important parasite in Latin America, infecting 10 million people.^1^ Throughout endemic regions, it is a leading cause of premature heart disease. The parasite is spread primarily by blood-sucking triatomine bugs, although other means of transmission include the congenital route, contaminated food and drink, organ transplantation, and blood transfusion. Due to increased mobility and migration, the disease is also appearing in nonendemic regions. There are estimated to be 300,000 infected individuals in the United States^2^ and 80,000 in Europe.^3^

Chagas disease can be divided into distinct stages. The “acute” stage is usually relatively mild and often undiagnosed. In children, the infection can be more severe, presenting as a febrile illness, with death from meningoencephalitis or myocarditis in up to 5% of diagnosed cases. With the development of an adaptive immune response, parasitemia is suppressed, although sterile immunity is not achieved. This “indeterminate” stage is asymptomatic and can be lifelong, with parasites difficult to detect. Around 30% of infected individuals proceed to the “chronic” stage, sometimes decades later.^4^ This phase is characterized by clinical manifestations, including cardiomyopathy and, more rarely, damage to the digestive tract (mainly megacolon and megaesophagus) and/or lesions in the peripheral nervous system.

*T. cruzi* is an obligate intracellular parasite and can invade many mammalian cell types, including smooth and cardiac muscle cells, macrophages, and neurons. Both innate and adaptive immune responses are activated by infection.^5–7^ Chagas disease is also characterized by induction of nonspecific polyclonal B-cell and T-cell responses, some of which may be autoreactive.^8,9^ These findings have prompted a longstanding debate about whether chronic stage cardiac disease results from autoimmune mechanisms or from inflammatory immune responses driven by parasite persistence within the heart and other organs.^10–13^

Because of the long-term nature of Chagas disease and its complex pathology, there is no realistic prospect of a vaccine in the foreseeable future. For treatment of *T. cruzi* infections, only two drugs are currently available, the nitroheterocyclic compounds nifurtimox and benznidazole.^14^ In each case, however, long-term therapeutic schedules are required, and toxic side effects are frequently observed. Although effective against acute stage infections, their ability to prevent the development of chronic chagasic heart disease is unresolved.^14,15^ Both nifurtimox and benznidazole are prodrugs and are activated by the same mitochondrial nitroreductase,^16,17^ a phenomenon with potential to result in cross-resistance. This, coupled with frequent reports of treatment failure, makes the development of new drugs a major priority.

## Bioluminescence Imaging

It has been almost a decade since noninvasive bioluminescence imaging technology was first used as a tool for studying protozoan infections.^18^ The approach requires the generation of genetically modified parasites capable of expressing luciferase.^19,20^ This class of enzyme oxidizes a luciferin substrate by an adenosine triphosphate (ATP)–dependent mechanism, resulting in light emission. Using an appropriate in vivo imaging system, parasite location within the infected host (almost invariably a mouse) can then be inferred and monitored in real time by detection of the signal. This procedure has been a powerful addition to the range of techniques applicable to infectious disease research.^21^ Importantly, whole-body bioluminescence can also be directly correlated with parasite load and provides a simple, noninvasive method for quantifying therapeutic intervention. Moreover, the ability to serially evaluate individual animals provides richer and more relevant data sets, while simultaneously enabling a drastic reduction in the number of animals required.

Most of the bioluminescence imaging systems applied to trypanosomatids^18,22–33^ have used reporter enzymes, such as firefly luciferase, which emit light in the blue-green region of the spectrum. One factor that has restricted their wider utility is the significant reduction in optical resolution that occurs at this wavelength (<600 nm) when parasites are situated deep within the viscera.^21^ This loss of sensitivity results from absorption and scattering of the emitted light. In the case of the former, hemoglobin is the primary chromophore, with absorbance occurring mainly in the region of the spectrum that overlaps with the light emitted in the luciferase reactions. With light scatter, the effect is also greater at shorter wavelengths. In combination, these factors result in blue-green light having restricted tissue penetration. To overcome these optical limitations, bioluminescent reporters that emit light above 600 nm have been engineered for in vivo imaging.^34,35^ As an example of the enhanced level of sensitivity that is now achievable, we have generated transgenic African trypanosomes expressing a red-shifted firefly luciferase (emission maximum changed from 562 nm to 617 nm), where the limit of detection is less than 100 parasites in an inoculated mouse.^36,37^ This is an improvement of up to three orders of magnitude on previously described reporter systems.

## Optimizing In Vivo Imaging of *T. cruzi* Infections

Several murine models that mimic aspects of human Chagas disease are available and have been widely used.^38–41^ However, predictive experimental systems that enable disease pathology to be matched to both the level and distribution of parasite burden during drug treatment have been lacking. This has limited our ability to identify the determinants of pathology and to assess drug treatment in real time. In chronic *T. cruzi* infection models, parasitemia is generally subpatent, and nondividing extracellular trypomastigotes are only rarely detected in the blood by standard microscopy procedures. One of our aims, therefore, was to develop an experimental model that would allow parasite dissemination and tissue tropism to be monitored in a noninvasive manner throughout chronic stage infections.

**Figure 1.**
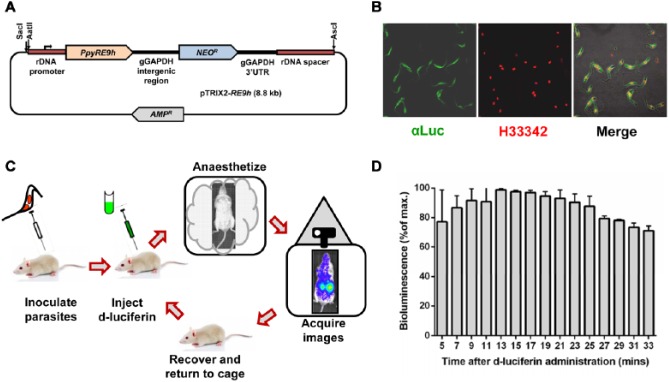
In vivo imaging of *Trypanosoma cruzi*. (**A**) The vector pTRIX2-RE9h^44^ was constructed by inserting the red-shifted firefly luciferase variant gene *PpyRE9h*^35^ into the *T. cruzi* rDNA targeting plasmid pTRIX.^50^ For transfection, the linearized targeting fragment can be produced by digesting the vector with the restriction enzymes indicated by arrows. (**B**) Cloned pTRIX2-RE9h–transfected *T. cruzi* epimastigotes, paraformaldehyde fixed and stained with anti–luciferase polyclonal antibody. Parasite DNA is stained red with Hoechst 33342. (**C**) Standard procedure for in vivo imaging experiments. Mice can be infected with bioluminescent parasites by the intraperitoneal, intravenous, or subcutaneous routes. At defined time points thereafter, they are inoculated with 150 mg^–1^ kg^–1^ d-luciferin, anaesthetized, and imaged for up to 5 min using an IVIS Lumina II system (PerkinElmer, Seer Green, UK).^44^ (**D**) Establishing the optimal “imaging window.” The variation in total ventral bioluminescence flux over time following injection of the luciferin substrate is shown.

First, we designed a DNA construct to integrate the *PpyRE9h* thermostable red-shifted luciferase gene^35,36^ into the multicopy ribosomal locus of the parasite, so that transcription would be under the control of an RNA polymerase I–dependent promoter and its associated downstream termination sequences^42^ (**Fig. 1A**). Trypanosomes are unusual in allowing integrated protein coding genes to be expressed in this context, and previous studies had demonstrated that high levels of transcription are achievable from ribosomal loci.^43^ When transgenic parasites were subsequently analyzed, we found considerable variation in the level of luciferase expression between different clones selected from the same transfection, a phenomenon that may be associated with locus-specific epigenetic regulation. However, once highly expressing parasite clones were isolated, the level of bioluminescence remained consistent between parasites in the population (**Fig. 1B**) and was stable over time. Parasites maintained for more than 300 days in absence of G418, the drug used to select transfectants, displayed unaltered levels of bioluminescence.^44^ In addition, there was no significant variation in the level of expression between the differing life cycle stages. Importantly, these parasites displayed growth and virulence properties that were indistinguishable from the parental CL Brener strain.

**Figure 2.**
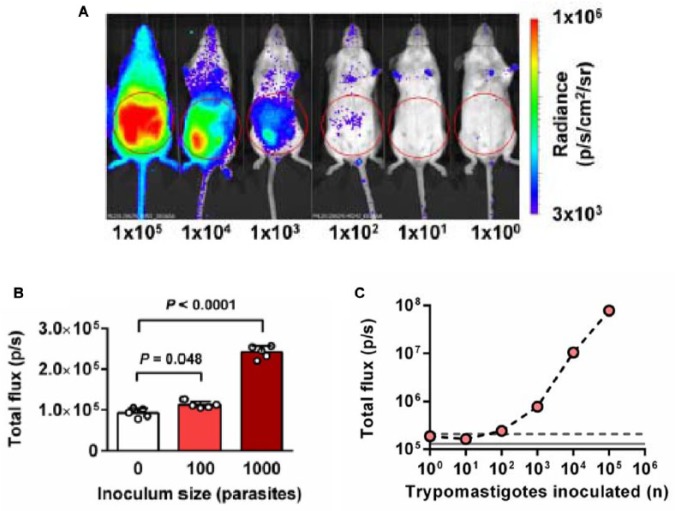
Highly sensitive in vivo imaging achieved with red-shifted luciferase. (**A**) Ventral images of SCID mice 1 h after intraperitoneal injection with bioluminescent bloodstream trypomastigotes (CL Brener strain, numbers indicated). The bioluminescence intensity is based on a log_10_ scale heatmap as indicated (right). (**B**) Abdominal bioluminescence displayed by mice 1 h after infection with 100 or 1000 bloodstream trypomastigotes. The regions used for signal quantification are outlined by the red circles in **A**. (**C**) Quantification of abdominal bioluminescence for mice shown in **A**. Dotted line represents background +2 SD. Figure adapted from Lewis et al.^44^

For in vivo imaging experiments, we normally inoculate mice by the intraperitoneal (i.p.) route, using bloodstream trypomastigotes derived from infected immunodeficient SCID mice. At defined time points thereafter, mice are injected with d-luciferin (**Fig. 1C**) and imaged 10 to 20 min later (**Fig. 1D**). Under these conditions, the limit of detection by in vivo imaging is close to 100 parasites (**Fig. 2A,B**), with a linear relationship between parasite load and whole-body bioluminescence at 1000 parasites and above^44^ (**Fig. 2C**). To put this into context, a single infected mammalian cell can contain several hundred amastigotes.

## In Vivo Imaging Provides New Insights into Chronic *T. cruzi* Infections

**Figure 3.**
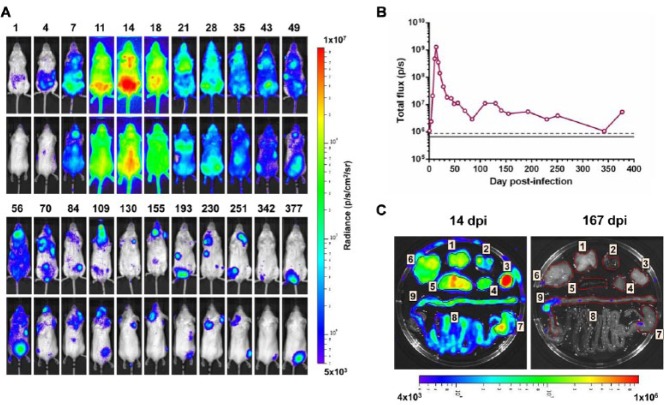
Imaging the development of chronic *Trypanosoma cruzi* infections. (**A**) Ventral and dorsal images of the same individual BALB/c mouse infected with *PpyRE9h*-expressing *T. cruzi* imaged at the indicated day postinfection (dpi) over the course of >1 year. (**B**) Quantification of combined ventral and dorsal bioluminescence for the mouse shown in **A**. Gray line and dashed line are the mean and mean +2 SDs, respectively, for uninfected control mice. (**C**) Ex vivo bioluminescence analysis of selected organs and tissue samples imaged immediately postmortem. An example is shown for acute (14 dpi) and chronic (167 dpi) infection. 1, lung; 2, heart; 3, visceral adipose; 4, skeletal muscle (quadriceps); 5, spleen; 6, gut mesenteries; 7, stomach; 8, small intestine; 9, large intestine.

With this bioluminescence imaging system, which is at least 10^2^-fold more sensitive than others reported previously,^22,26,28,33^ it was possible for the first time to track *T. cruzi* distribution in an individual mouse for more than a year throughout a long-term chronic infection (**Fig. 3A,B**). In the BALB/c model, following i.p. inoculation with 10^3^ bloodstream trypomastigotes (CL Brener strain), total bioluminescence peaked 14 days postinfection, with parasites widely disseminated, infecting all of the major organs, including the heart (**Fig. 3C**). The ubiquitous detection of bioluminescence, which could be correlated with parasite burden by tissue-specific quantitative PCR (qPCR),^44^ suggests that the bioavailability of d-luciferin is not a limiting factor in application of the technology. This peak in parasite burden during the acute stage was followed by a progressive decrease and elimination from most tissues, with parasites microscopically undetectable in blood smears by day 35.^44^ As infections entered the chronic stage, total parasite levels were up to 1000 times lower than in the acute stage, although highly dynamic bioluminescent foci could be detected that fluctuated in a spatiotemporally dynamic manner (**Fig. 3A,B**). Typically during the chronic stage, between 1 and 5 specific infection foci could be observed in each animal, with these appearing and disappearing over a 24- to 48-h period. This pattern was independent of the route of inoculation (i.p., intravenous [i.v.], or subcutaneous [s.c.]) or the type of infectious parasite used to initiate the infection (metacyclic, bloodstream-form, or tissue culture–derived trypomastigotes). Given the short-term nature of these bioluminescent foci and the sensitivity of the imaging system, the most likely explanation is that they represent single infected phagocytes that become detectable as they are trafficked to peripheral sites.

To gain a more definitive assessment of tissue-specific parasite burdens, ex vivo imaging of selected organs and tissue samples can be undertaken immediately postmortem. When BALB/c mice in the chronic stage of infection were analyzed using this approach, parasites were consistently found to be restricted to the gastrointestinal tract (GIT), mainly localized to the colon and/or stomach (**Fig. 3C**). Minor infection foci were detected in the gut mesenteries or skeletal muscle on some occasions but surprisingly not in the heart, the main site of disease pathology. Despite the absence of locally persistent heart infection in this model, mice displayed signatures of chagasic cardiac pathology, including infiltration with inflammatory mononuclear cells, mild edema, and fibrosis.^44^ The absence of parasites in heart tissue and their persistent presence in the GIT were confirmed by a highly sensitive qPCR-based DNA detection assay.

These observations provide new insights into *T. cruzi* infection and the processes underlying disease pathology. First, they implicate the GIT as a permissive niche that acts as a parasite reservoir, allowing long-term parasite persistence. Second, they suggest a mechanism that facilitates disease transmission by ensuring continual migration of parasite-infected cells to peripheral sites where they become accessible to triatomine vectors during a blood meal. Third, they provide evidence that locally persistent infection of the heart during chronic infection is not an essential requirement for the development of cardiac pathology during chronic *T. cruzi* infections.

## An Improved Experimental Model for Assessing Drug Efficacy

Attempts to assess the efficacy of new drugs against Chagas disease are made complex by the long-term nature of the disease in humans (which can stretch over decades) and difficulties in demonstrating parasitological cure during the chronic stage. In experimental animal models, continuous monitoring of parasite burden is also difficult to achieve because bloodstream infections are generally subpatent and tissue sampling requires animals to be sacrificed at each time point. While PCR-based methodologies benefit from high sensitivity, reproducibility has often been a problem. This is now understandable given the discrete locations of infection foci and their highly dynamic nature (**Fig. 3**). The advances in imaging technology described above therefore provide a basis for developing improved predictive models for drug development programs. This can be demonstrated by monitoring the effectiveness of benznidazole therapy against both acute and chronic infections.

**Figure 4.**
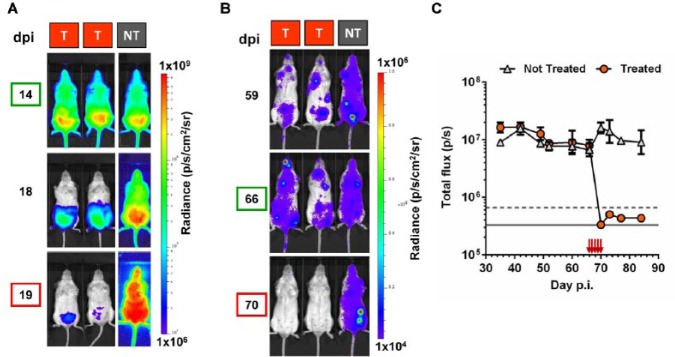
Validation of in vivo imaging as a tool to assess efficacy of drug treatment against acute and chronic *Trypanosoma cruzi* infections. (**A**) SCID mice infected with 10^3^ bioluminescent *T. cruzi* bloodstream trypomastigotes (CL Brener strain) were treated (T) with benznidazole (daily by oral route, 100 mg^–1^/kg^–1^) 14 to 19 days postinfection (dpi) and compared with control nontreated (NT) mice. The bioluminescence intensity is represented by a log_10_ scale heatmap as indicated (right). (**B**) BALB/c mice chronically infected with bioluminescent *T. cruzi* were treated (T) for 5 days with 100 mg^–1^/kg^–1^ benznidazole by the oral route starting at 66 dpi and compared with control nontreated (NT) mice. The bioluminescence intensity is represented by a linear scale heatmap as indicated (right). (**C**) Quantification of total ventral bioluminescence for the experiment including the mice shown in **B** (*n* = 3). Gray line and dashed line are the mean and mean +2 SDs, respectively, for uninfected control mice. Red arrows indicate treatment days.

When immunodeficient SCID mice are inoculated with *T. cruzi*, there is a widely disseminated infection (**Fig. 4A**) that causes a rapidly progressive cachexia-like syndrome. Humane end points are typically reached within 3 to 4 weeks depending on the inoculum. We treated mice with oral benznidazole (100 mg/kg^–1^), starting 14 days postinfection (**Fig. 4A**), and observed a progressive reduction in parasitemia over the 5-day treatment schedule, as assessed by in vivo imaging. This correlated with improvements in animal condition and the reversion of parasitemia to subpatent levels. However, when mice were first judged as clear by microscopic examination of peripheral blood samples, parasites remained readily detectable by bioluminescence (e.g., day 18; **Fig. 4A**). Similarly, the sensitivity of the in vivo imaging system has provided a means of monitoring the effectiveness of therapy against chronic *T. cruzi* infections in an experimental model of Chagas disease, when parasites are rarely visualized in the bloodstream. As a shown in **Figure 4B,C**, the approach allows the real-time evaluation of treatment in BALB/c mice in a manner that should be scalable to the needs of a drug development pipeline.

The most challenging aspect for the development of predictive treatment models, and one that is by no means unique to Chagas disease research, is the capacity to unequivocally demonstrate sterile cure. In the absence of definitive cure criteria, a number of proxy measures are commonly used, including fresh blood examination, blood cultures, inoculation of immunocompromised animals with blood samples, PCR on DNA from blood and tissue samples, and serological assays. Tissue PCR is understandably the most sensitive of these traditional approaches and can identify a significant number of noncured mice that would otherwise appear as cured on blood-based criteria.^45^ The insight into the dynamics of *T. cruzi* infection provided by bioluminescence imaging shows that even tissue PCR is likely to misclassify treatment failures as cures, due to the highly focal distribution of parasites. The immunosuppressive drug cyclophosphamide has been used to enhance posttreatment parasite detection sensitivity,^46–48^ the rationale for this being that residual foci of infection are allowed to expand to detectable levels when adaptive immunity is compromised. Thus, combining cyclophosphamide treatment with bioluminescence imaging should provide a maximally sensitive approach to assess cure and relapse.

**Figure 5.**
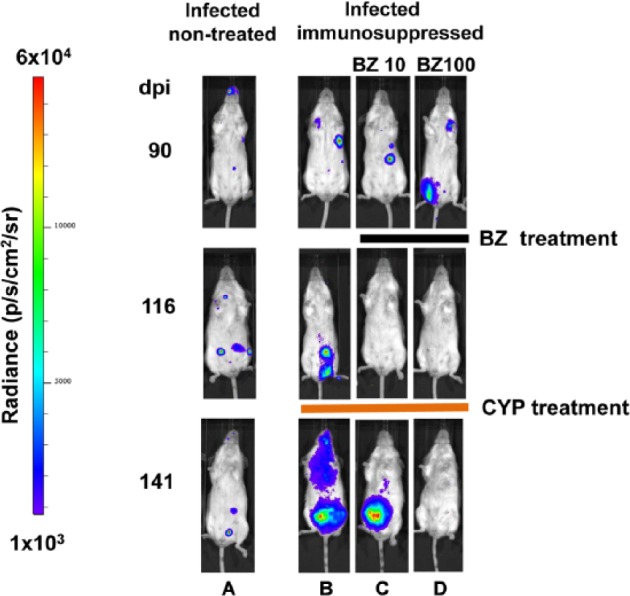
Combining in vivo imaging and immunosuppression to monitor the effectiveness of benznidazole treatment. Ventral views of BALB/c mice infected with 10^3^ bioluminescent *Trypanosoma cruzi* bloodstream trypomastigotes and treated with benznidazole. (**A**) Nontreated control. (**B**) Nontreated, immunosuppressed with 200 mg-^1^ kg^-1^ cyclophosphamide by intraperitoneal injection 124, 127, 130, and 133 days postinfection (dpi). (**C**) Treated with daily oral doses of 10 mg^-1^ kg^-1^ benznidazole between 90 and 110 dpi, then immunosuppressed with cyclophosphamide as above. (**D**) Treated with daily oral doses of 100 mg^-1^ kg^-1^ benznidazole between 90 and 110 dpi, then immunosuppressed with cyclophosphamide. The bioluminescence intensity is represented by a linear scale heatmap as indicated (left).

Cyclophosphamide has toxic side effects and increases the risk of secondary infections, so it is important to implement appropriate monitoring procedures. It is also necessary to tailor immunosuppression protocols to particular host-parasite strain combinations. For example, two alternative treatment schedules have been described.^46,49^ Preliminary testing in our laboratory on BALB/c mice chronically infected with the *PpyRE9h*-expressing CL Brener strain has shown that immunosuppression does lead to an increase in bioluminescence (**Fig. 5**), although neither protocol caused reversion to patent parasitemia. We were also able to demonstrate that treatment with 100 mg-1 kg-1 benznidazole daily for 20 days results in sterile cure, whereas treatment with 10 mg^-1^ kg^-1^ does not, even though parasite burden falls transiently below the level of detection (**Fig. 5** as an example). Integrating posttreatment immunosuppression and bioluminescence imaging should therefore provide a method to evaluate the true dynamics of *T. cruzi* reactivation and dissemination in animals that are treated but not cured.

In conclusion, drug development for Chagas disease has been hampered by a lack of sufficiently sensitive tools for the detection of parasites that remain following drug treatment of experimental infections. Highly sensitive in vivo imaging models using transgenic bioluminescent *T. cruzi* parasites therefore represent an important advance. This technology is reproducible, is relatively scalable, and can be adapted to diverse experimental schedules and models. Furthermore, serial imaging of individual animals means the data generated are more robust yet require far fewer experimental animals than with traditional methods. It is important to recognize that bioluminescence imaging alone will not provide a definitive measure of sterile cure, but when combined with posttreatment immunosuppression, it will provide a drastically improved approach to evaluate the efficacy of novel therapeutic compounds for the treatment of *T. cruzi* infections.
